# Supplementary material for: Predictors of recurrence of major depressive disorder
Source: PLoS One. 2020 Mar 19;15(3):e0230363. doi: 10.1371/journal.pone.0230363 (PMC7082055; doi:10.1371/journal.pone.0230363)
Supplement: S1 Appendix — (DOCX) [file pone.0230363.s001.docx]

**CLINCAL RECORD FORM***

**RECURRENCE OF MAJOR DEPRESSIVE EPISODE ID __________**

| **No.** | **Clinical History** | **Response** |  |  |  |
| --- | --- | --- | --- | --- | --- |
| R1 | Family History of Major Depressive Disorder | - Yes - No |  |  |  |
| R2 | Date of diagnosis of FIRST Major Depressive Episode | _ _ _ _ _ _ _ _ DD MM YYYY |  |  |  |
| R3 | R3j. Severity of FIRST Major Depressive Episode | - Mild - Moderate - Severe | - R3a. Depressed mood (subjective or observed) - R3b. Loss of interest or pleasure in most daily activities - R3c. Thoughts of death, suicidal ideation or suicidal attempt - R3d. Inappropriate guilt or sense of worthlessness | - R3e. Change in weight or appetite - R3f. Insomnia or hypersomnia - R3g. Loss of energy or fatigue | - R3h. Impaired concentration or indecisiveness - R3i. Psychomotor agitation or retardation (observed) |
| R4a | Symptoms of MDE in the past 2 months after date of diagnosis | - No significant sign or symptom of MDD in the past 2 months - Symptoms of immediate previous MDE are present but do not fulfill full criteria of MDD - Failure to respond to two or more anti-depressant treatment (150mg/day of imipramine) | | | |
| R4b | Status of remission from FIRST Major Depressive Episode | - Partial remission - Full remission - Treatment resistant MDD | | | |
| R4c | Partial remission from 1^st^ MDE | - Yes - No | | | |
| R4d | Date of achieving partial remission from FIRST MDE | _ _ _ _ _ _ _ _  DD MM YYYY | | | |
| R4e | Duration from FIRST MDE to Partial Remission | ____________________ days | | | |
| R4f | Full remission from First MDE | - Yes - No | | | |
| R4g | Date of Achieving Full Remission from FIRST MDE | _ _ _ _ _ _ _ _  DD MM YYYY | | | |
| R4h | Duration from 1st MDE to Full Remission | _________ | | | |
| R5a | Date of 1st RECURRENCE | _ _ _ _ _ _ _ _  DD MM YYYY | | | |
| R5b | Duration of 1st Recurrence from 1st MDE | ____________________ days | | | |
| R5l | Severity of First Recurrence | 1 Mild  2 Moderate  3 Severe | - R5c. Depressed mood (subjective or observed) - R5d. Loss of interest or pleasure in most daily activities - R5e. Thoughts of death, suicidal ideation or suicidal attempt - R5f. Inappropriate guilt or sense of worthlessness | - R5g. Change in weight or appetite - R5h. Insomnia or hypersomnia - R5i. Loss of energy or fatigue | - R5j. Impaired concentration or indecisiveness - R5k. Psychomotor agitation or retardation (observed) |
| R6a | Symptoms of MDE in the past 2 months after first recurrence | - No significant sign or symptom of MDD in the past 2 months - symptoms of immediate previous MDE are present but do not fulfill full criteria of MDD - failure to respond to two or more anti-depressant treatment (150mg/day of imipramine) | | | |
| R6b | Status of Remission from First Recurrence | - Partial remission - Full remission - Treatment resistant MDD | | | |
| R6c | Partial Remission from First Recurrence | - Yes - No | | | |
| R6d | Date of Achieving Partial Remission from 1st Recurrence | _ _ _ _ _ _ _ _  DD MM YYYY | | | |
| R6e | Duration from 1st Recurrence to Partial Remission |  | | | |
| R6f | Full Remission from 1st Recurrence | - Yes - No | | | |
| R6g | Date of Achieving Full Remission from 1st Recurrence | _ _ _ _ _ _ _ _ DD MM YYYY | | | |
| R6h | Duration from 1st Recurrence to Full Remission |  | | | |
| R7a | Date of 2nd RECURRENCE | _ _ _ _ _ _ _ _ DD MM YYYY | | | |
| R7b | Duration of 2^nd^ RECURRENCE from previous MDE |  | | | |
| R7l | Severity of FIRST RECURRENT major depressive episode | - Mild - Moderate - Severe | - R7c. Depressed mood (subjective or observed) - R7d. Loss of interest or pleasure in most daily activities - R7e. Thoughts of death, suicidal ideation or suicidal attempt - R7f. Inappropriate guilt or sense of worthlessness | - R7g. Change in weight or appetite - R7h. Insomnia or hypersomnia - R7i. Loss of energy or fatigue | - R7j. Impaired concentration or indecisiveness - R7k.Psychomotor agitation or retardation (observed) |
| R8a | Symptoms of MDE in the past 2 months after 2nd recurrence | - No significant sign or symptom of MDD in the past 2 months - Symptoms of immediate previous MDE are present but do not fulfill full criteria of MDD - Failure to respond to two or more anti-depressant treatment (150mg/day of imipramine) | | | |
| R8b | Status of Remission from 2nd Recurrence | - Partial remission - Full remission - Treatment resistant MDD | | | |
| R8c | Partial Remission from 2nd Recurrence | - Yes - No | | | |
| R8d | Date of Achieving Partial Remission from 2nd Recurrence | _ _ _ _ _ _ _ _ DD MM YYYY | | | |
| R8e | Duration from 2nd Recurrence to Partial Remission | ____________________ days | | | |
| R8f | Full Remission from 2nd Recurrence | - Yes - No | | | |
| R8g | Date of Achieving Full Remission from 2nd Recurrence | _ _ _ _ _ _ _ _ DD MM YYYY | | | |
| R8h | Duration from 2nd Recurrence to Full Remission | ____________________ days | | | |
| R9 | Number of Recurrences up to 30th April 2018 | ____________________ days | | | |
| R10 | Date Censored | _ _ _ _ _ _ _ _ DD MM YYYY | | | |

*information extracted from case notes
