# Supplementary material for: Predictors of recurrence of major depressive disorder
Source: PLoS One. 2020 Mar 19;15(3):e0230363. doi: 10.1371/journal.pone.0230363 (PMC7082055; doi:10.1371/journal.pone.0230363)
Supplement: S2 Appendix — (DOCX) [file pone.0230363.s002.docx]

****S2 Appendix. Genotyping of VDR Single Nucleotide Polymorphisms (SNPs)****

Polymerase chain reaction-restriction fragment length polymorphism (PCR-RFLP) was used to genotype the three VDR SNPs, *Bsm*I (rs1544410), *Apa*I (rs7975232) and *Taq*I (rs731236) at the Department of Biomedical Sciences Laboratory, Universiti Putra Malaysia. Oligonucleotide primers (listed in Table 1) were used to amplify a region of 1,930 bp carrying the polymorphic restriction sites of *Bsm*I (rs1544410), *Apa*I (rs7975232) and *Taq*I (rs731236). Amplification was performed in a total volume of 50 μL PCR reaction mixture containing 100 ng of genomic DNA.

Table 1. Sequence of forward (F) and reverse (R) primers in *VDR* genotyping.

| **Sequence 5’- 3’** | **Length**  **(Basepairs)** | **Amplified fragment (Basepairs)** |
| --- | --- | --- |
| F-CAACCAAGACTACAAGGTACCGGCGGTCAGTGGA | 30 | 1,930 |
| R-CACTTCGGAGCACAAGGGGGGCGTTAGC | 25 |  |

PCR composition, PCR condition and RFLP digestion of *Bsm*I, *Apa*I and *Taq*I polymorphisms.

50 μL of total PCR reaction includes 25 μL of 2X DreamTaq green PCR mastermix consisting of DreamTaq DNA polymerase, 2X DreamTaq Green buffer, dATP (0.4 mM), dCTP (0.4 mM), dGTP (0.4 mM) and dTTP (0.4 mM), and 4 mM MgCl_2_ (Thermo Fisher Scientific, MA, USA), 1 μL of each primers (10 μM), 1 μL of genomic DNA (100ng/µL) and 22 μL of nuclease free water.

PCR condition was as follows: 94°C for 4 minutes, followed by 35 cycles of denaturation at 94°C for 30 seconds, annealing at the melting temperature 59°C for 45 seconds and extension at 72°C for 45 seconds, with a final 10 minutes extension at 72°C.

For RFLP digestion of *BsmI*, 10 μL of PCR product was digested by 1 μL of *Bsm*I restriction endonuclease (10U/ μL) (New England Biolabs, MA, UK) at 65^o^C for 3 hours. For RFLP digestion of *ApaI*, 10 μL of PCR product was digested by 1 μL of *ApaI* restriction endonuclease (50U/μL) (New England Biolabs, MA, UK) at 37^o^C for 4 hours. And for *TaqI,* 10 μL of PCR product was digested by 1 μL of *Taq^α^*I restriction endonuclease (20U/ μL) (New England Biolabs, MA, UK) at 65^o^C for 4 hours.

Restriction fragment length polymorphism analysis was performed on the amplified fragments. The T/T (BB) genotype produced a single undigested product with 1930 bp, the C/T (bB) genotype produced partially digested 1930 bp, 1285 bp and 645 bp fragments, the C/C (bb) genotype was fully digested into 1285 bp and 645 bp fragments, using the restriction endonucleases *Bsm*I. Digestion of the PCR product with *Apa*I restriction enzyme resulted in an undigested 1930 bp product for A/A (AA) genotype. C/A (aA) genotype was partially digested into 1930 bp, 1643 bp and 287 bp fragments while C/C (aa) genotype underwent full digestion into 1643 bp and 287 bp fragments. For *TaqI* polymorphism, the genotype T/T (TT) produced undigested 1930 bp fragment, the genotype T/C (Tt) was partially digested into 1930 bp, 1723 bp and 207 bp fragments, and fully digested C/C (tt) genotype produced 1723 bp and 207 bp fragments. 10% of PCR product were sent to First Base Laboratories Sdn. Bhd., Malaysia for Sanger sequencing service. The Codon-Code Aligner software (CodonCode Corporation, MA, USA) was used for sequence assembly, contigs generation and contigs alignment. The CLUSTAL algorithm was used for contigs alignment. Quality of sequencing was assessed using Phred scores. Scores of more than 20 was considered good quality sequencing^1^.

Reference:

1. Mbandi SK, Hesse U, Rees DJG, Christoffels A. A glance at quality score: implication for denovo transcriptomere construction of Illumina reads. Front Genet. 2014; 5 (12): 17.
