# Supplementary material for: Predictors of recurrence of major depressive disorder
Source: PLoS One. 2020 Mar 19;15(3):e0230363. doi: 10.1371/journal.pone.0230363 (PMC7082055; doi:10.1371/journal.pone.0230363)
Supplement: S3 Appendix — (DOCX) [file pone.0230363.s003.docx]

**S3 Appendix. Genotyping for single nucleotide polymorphisms of *HTR1A*-rs6295 and *HTR2A*-rs6311**

For polymerase chain reactions (PCR), a total reaction volume of 25μl with a final reaction concentration of 0.4 μM forward (5’ TGG AAG AAG ACC GAG TGT GTC TAC 3’) and 0.4μM reverse (5’ TTC TCC CTG AGG GAG TAA GGC TGG 3’) primers for *HTR1A*rs6295 polymorphism for the polymerase chain reactions (PCR). For the 5HTR2A gene polymorphism, the final concentration of 0.4 μM was also used for both forward (5’CTGGGTGGCATATTCTGCT 3’) and reverse (5’ ACC AAG GGA CTCCTGGTTTC 3’) primers. The reaction mix also contains a 1x Top taq mastermix (Qiagen) final reaction concentration consisting of 1.25 units of Top Taq DNA polymerase and 200 μM of each dNTPs. Genomic DNA of >1μg was also added to the reaction mix and the total reaction volume was made up to the 25μl using RNAse free DNA water.

For the PCR of *HTR1A*-rs6295 polymorphism, the initial denaturation was 95°C for 3 minutes, and each cycle had a denaturation temperature at 95°C for 45 seconds, annealing was at 56°C for 45 seconds and extension was at 72°C for 45 seconds. For the *HTR1A*-rs6295 polymorphism, a total of 36 cycles were carried out with a final extension set at 72°C for 10 minutes^1^. For the PCR of *HTR2A*-rs6311 polymorphism, the initial denaturation was 94°C for 3 minutes, each cycle had a denaturation temperature at 94°C for 60 seconds, annealing was at 56°C for 60 seconds and extension was at 72°C for 60 seconds. A total of 35 cycles were carried out with a final extension set at 72°C for 9 minutes (Kim et al., 2008).

For purification and DNA sequencing, all PCR products were sent to First BASE Laboratories Sdn Bhd (Malaysia), which used the ABI PRISM xl3730 DNA Analyzer and BigDye Terminator Cycle Sequencing Kit. All sequencing results were blasted to verify the sequences with those from the NCBI data base and genotypes were determined using DNA BASER software tool.

References:

1. Choi WS, Lee BH, Yang JC, Kim YK. Association Study between 5-HT1A Receptor Gene C(-1019)G Polymorphism and Panic Disorder in a Korean Population. Psychiatry Investig. 2010;7(2):141-6.
2. Kim KH, Woo HY, Lim SW. Association Study of a Serotonin Receptor 2A Gene -1438A/G Polymorphism and Anxiety-Related Traits. Psychiatry Investig. 2008; 5(4): 244 – 246.
